# Ranking of meal preferences and interactions with demographic characteristics: a discrete choice experiment in young adults

**DOI:** 10.1186/s12966-020-01059-7

**Published:** 2020-12-01

**Authors:** Katherine M. Livingstone, Karen E. Lamb, Gavin Abbott, Tony Worsley, Sarah A. McNaughton

**Affiliations:** 1 Institute for Physical Activity and Nutrition (IPAN), School of Exercise and Nutrition Sciences, Deakin University, Geelong, Victoria Australia; 2 Melbourne School of Population and Global Health, University of Melbourne, Parkville, Australia

**Keywords:** Discrete choice experiment, Food preferences, Meal preferences, Barriers, Healthy eating, Dietary patterns, Eating behaviours, Young adults, Online survey

## Abstract

**Background:**

The diet of young adults is poor, yet little is known about the relative importance of influences on healthy eating in a decision-making context. The aim of this exploratory study was to understand the relative ranking of influences on meal choices in young adults and to investigate interactions between meal preferences and demographic and health characteristics.

**Methods:**

Adults aged 18–30 years (*n* = 92, mean age: 23.9 (SD 3.4) years) completed an online discrete choice experiment. Participants were presented with 12 choice sets reflecting a typical weekday meal and were asked to choose between four meal options. Each meal consisted of a combination of five meal attributes (preparation time, cost, taste, familiarity and nutrition content) that each had three attribute levels. Data were analysed using conditional logit models. Subgroup analyses were performed by sex, education, income, weight status and meeting fruit and vegetable recommendations.

**Results:**

Comparing the highest and lowest attribute levels, meal preferences were higher for better taste (B = 0.38; 95% CI: 0.12, 0.63), familiarity (B = 0.37; 95% CI: 0.21, 0.54) and nutrition content (B = 1.11; 95% CI: 0.81, 1.41) and lower for increased preparation times (B = −0.33; 95% CI: − 0.53, − 0.12) and cost (B = −0.50; 95% CI: − 0.75, − 0.24). Nutrition content was the most important influence on meal choice. Cost was the second most important, followed by taste, familiarity and preparation time. Compared to males, females had a higher preference for better nutrition content, taste and familiarity and a lower preference for increased cost. Higher educated participants had a higher preference for better nutrition content, familiarity and taste compared to lower educated participants. Young adults who met recommendations for fruit and vegetable intake had a higher preference for better nutrition content compared to participants who did not meet recommendations.

**Conclusion:**

Nutrition content was the most important influence on young adults’ meal choices, followed by cost, taste, familiarity and preparation time. Preferences varied by demographics and health characteristics, suggesting that the focus of dietary interventions may benefit from being tailored to specific young adult groups.

## Introduction

Young adulthood is typically characterised as a period of multiple transitions, including commencing higher education, experiencing new working environments and starting a family [1]. These changes in social and financial circumstances can impact on health behaviours, such as dietary choices [2]. The diet of young Australian adults is poor, with less than 5% meeting recommend intakes of vegetables, and discretionary foods contributing up to 40% of total energy intake [3]. In addition, prevalence of meal skipping is at 20%, and unhealthy snacking and eating out are frequent occurrences [4]. To support the implementation and translation of policies and interventions aiming to improve dietary choices in young adults, a better understanding of what influences dietary behaviours in this population group is needed.

Commonly reported influences on young adults’ food choices include a lack of time to plan, shop, prepare and cook healthy foods, cost and the preferred taste and convenience of unhealthy foods [5, 6]. The relevance of these influences may also vary by young adults’ demographic characteristics, such as by sex [6, 7] and socioeconomic position [5, 6]. For example, there is evidence that young adult males are less interested in health, whereas females are more health-conscious, and that there is a lower perceived affordability of healthy foods in lower socioeconomic groups compared to higher socioeconomic groups [5, 8, 9].

Understanding of the influences on healthy eating decisions is limited by a reliance on methodologies that do not account for the complexity of food choice behaviours. Behavioural models, such as the theory of planned behaviour, have shown that food choice decisions are dependent on a complex combination of the type of food choice behaviour, the participants’ characteristics, their intention-behaviours and their perceived behavioural control [10]. Food choices thus involve trade-offs, requiring a methodology that captures this complexity. Discrete Choice Experiments (DCE) are quantitative techniques designed to identify and evaluate the relative importance of aspects of decision making [11]. DCEs overcome the limitations of traditional questionnaires for quantitatively ranking factors that influence dietary choices by forcing participants to make trade-offs between these influences.

While DCEs have been predominantly used in health economics for primary care treatment [12–15], their potential for use within the context of nutrition research is becoming increasingly recognised [9, 16–22]. DCEs offer an opportunity to identify the most important influences on meal-based eating decisions that should be targeted to improve dietary interventions and policies in young adults. However, DCE research on meal choices to date has focused on middle to older adults [19, 23], parents [22] and children [20]. To our knowledge, only one DCE has been undertaken in young adults, which focused on snacking behaviours in college students [21]. Attributes examined have been comparable between studies, although no DCEs to date have examined the role of familiarity when making meal decisions. This is potentially important in young adults given links between unfamiliar foods, food avoidance behaviours and poor diet quality in this age group and that young adults have the smallest number of meals in their repertoire [24]. Moreover, of the few studies that have examined how meal preferences vary by participant subgroups, all have focused on indicators of socio-economic position and have used face-to-face DCE methodologies and only female participants [23]. The application of an online DCE to explore meal preferences in a population-based sample of young adults is thus unknown. Thus, the primary aim of this study was to understand the influences on meal choices in young adults (18–30 years) and their relative ranking using an online DCE. The secondary aim was to investigate interactions between meal preferences and demographic and health characteristics.

## Methods

### Study design

Participants were recruited between November 2017 and December 2017 into the CHOICE study, an internet-based survey that included a DCE to understand dietary choices in young adults. This paper reports the study according to the STROBE-nut Checklist for reporting results from nutritional epidemiology (Additional File 1) [25]. The Deakin University Faculty of Health Human Research Advisory Group approved this study (152_2017) and all participants provided informed consent. Participant confidentiality was protected through the collection of non-identifiable data, which were stored on password-protected computers.

### Participants

With limited consensus on sample sizes for DCEs, a minimum sample size of *n* = 100 was considered sufficient to enable the modelling of preference data and utility levels for the primary outcome, as described by Pearmain et al. [26] A convenience population-based sample of young adults (aged 18–30 years) was recruited using sponsored advertisements on Facebook, Instagram and Twitter. The following inclusion criteria were used: 1) aged between 18 and 30 years; 2) living in Australia; 3) not currently pregnant or breastfeeding and 4) English as the primary language spoken at home.

### Online procedures

The internet-based survey and DCE were delivered via Sawtooth Software (Lighthouse Studio 9.4.0). Social media advertisements directed individuals to the open survey via a study-specific link hosted by Sawtooth. Potential participants were directed through to an online plain language statement, screening questions and online consent. The plain language statement included information on the aims of the CHOICE study, what was involved for participants, any risks and benefits and how their confidentiality would be protected. Interested individuals were screened to ensure that they met the eligibility criteria. Participants not meeting the inclusion criteria were excluded. Participation was voluntary and participants could withdraw at any time. Consenting participants were directed straight through to the online questionnaire. On completion, participants received a $10 e-gift voucher as compensation. The survey was open for 2 weeks, which would allow for the target of 100 participants to undertake the survey.

### Discrete choice experiment

An internet-based survey and DCE was preferable for use in young adults due to the high use of the internet and mobile devices in this age group [27, 28]. Online DCEs have been shown to be comparable to face-to-face DCEs [29]. In the DCE, participants were presented with choice sets. Choice sets are used to combine hypothetical alternatives based on a combination of attributes (characteristics), which in this case were meals representing a combination of meal attributes (e.g. meal cost) [11]. Meals were described to participants as a typical weekday meal at home, but were not restricted to a particular time of day. Within each choice set, participants were asked to choose a single alternative (i.e. a meal). This choice reflected their hypothetical, or stated preference [11]. As described by de Bekker-Grob et al., the DCE approach includes a number of theories: i) consumer theory, where any meal would be regarded as a given bundle of attributes and a combination of meals will produce a vector of quantities of these attributes [30]; ii) random utility theory, where participants would opt for the meal alternative with the highest utility (or value) [31]; iii) experimental design theory, where approaches to design an efficient and effective DCE are considered [32] and iv) econometric analysis, where different analysis models are available depending on the research question [13]. Choices reflected the underlying utility function. Three steps were involved in the development of the DCE used in this study: i) five attributes were chosen; ii) three attributes levels were specified; and iii) hypothetical alternatives of attribute levels were combined to create choice sets. Attributes and levels were identified based on review of the literature [9, 33], and were piloted in a convenience sample of staff (*n* = 5) prior to constructing the DCE. These choice sets were then shown to participants.

#### Experimental design

**Fig. 1:**
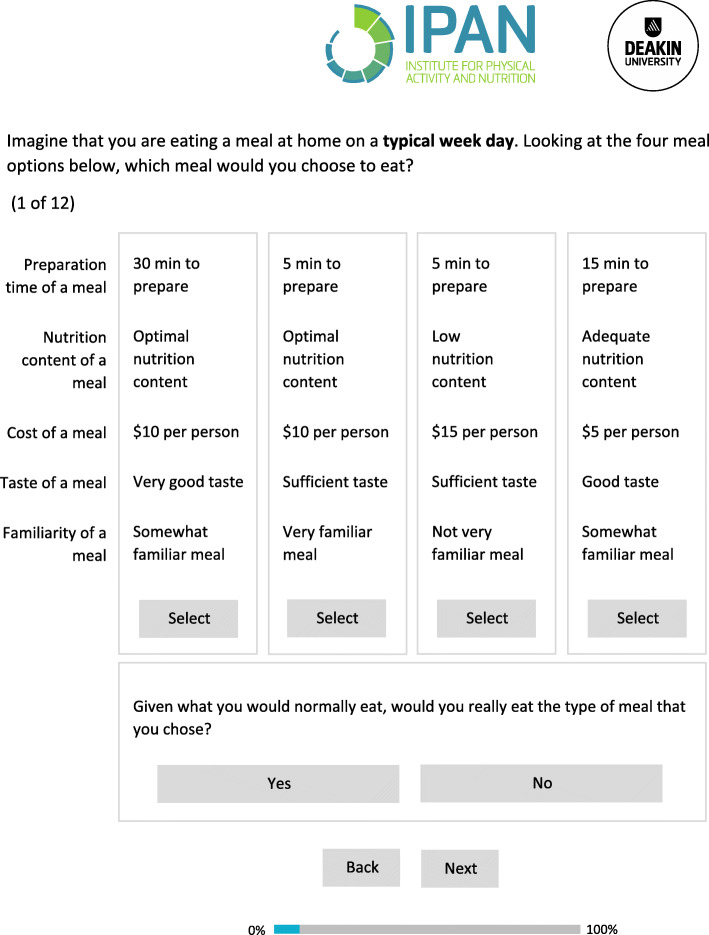
Example of a discrete choice experiment choice set used in the CHOICE Study

The DCE experimental design was created using Sawtooth Software. The final experimental design was identified based on an optimal design efficiency (using SE and relative D-efficiencies) and to minimize participant burden [11]. A total of 10 designs were created to identify an optimum design. These designs varied according to number of choice sets (8 to 14), meal alternatives (3 or 4 meals) and chosen random meal generation methods (balanced overlap or complete enumeration) [34]. The final design included five attributes with three levels, 12 choice sets per participant, four meal alternatives per choice set and a balanced overlap design (SE 0.43; relative D-efficiency 1.07). This experimental design combination resulted in 243 possible meal options. A balanced overlap design is a random task generation method available in DCE experimental design software to ensure that each attribute level within an attribute appears an equal number of times to participants [11]. We used this method as it ensured i) moderate overlap between meal attributes, ii) no duplicate meals permitted within the same choice set and iii) conditions for orthogonality (i.e. where each attribute level appears an equal number of times in combination with all other attribute levels) were well controlled [11]. As recommended by Sawtooth Software, the design was set to generate the default of 300 unique versions of the survey and a design seed of one was used. All attributes were randomized and meal sorting was designed so that level order was randomized. Including a “none” option in the choice set (i.e. a non-forced DCE) may better reflect participant choices given that individuals can choose to not eat a meal presented to them. However, including a “none” option as a parameter, or meal choice, in the DCE may result in a loss of precision in other parameters if a high proportion of respondents chose “None” [11]. As a result, a dual response option was chosen, which presented participants with a two-stage question about their meal choice. While the meal choice was forced, they were then presented with a question where they had the option of “opting out”. The “none” option was represented by the following question located below the meal options in the choice set “Given what you would normally eat, would you really eat the type of meal that you chose?”, where participants could respond “Yes” or “No” (Fig. 1).

#### Attributes and attribute levels

In accordance with DCE reporting guidelines [32], attributes were identified from the relevant literature. Findings from a recent systematic review identified that the most common correlates of unhealthy eating behaviours in young adults were perceived lack of time and cost [33]. A recent DCE in older adults showed that healthiness of a meal was the most important influence on meal preferences and that familiarity was an area where more research was needed [9]. Little is known about the ranking of healthiness and familiarity in dietary preferences of young adults [9]. Attribute levels for nutrition content, preparation time, taste and familiarity were based on previous literature and piloted in a convenience sample (*n* = 5) to ensure that the range of choices was plausible and understandable for respondents [9, 33]. Attributes and levels were as follows: Preparation time: 5 min (ready meals); 15 min; 30 min. Nutrition content: low; adequate; optimal. Cost: $5 per person; $10 per person; $15 per person. Taste: sufficient; good; very good. Familiarity: not very; somewhat; very. For the purpose of this study, nutrition content was based on the number and type of serves of vegetables in the meal. We classified low nutrition content as containing no vegetables, adequate as one serve of canned vegetables (with added sugar or salt), pickled vegetables or vegetables roasted in oil or marinated and optimal as one or more serves of fresh, frozen or canned vegetables (without any added salt or sugar). Distinct from previous research [9], this provided a quantitative reference based on meal-based healthy eating guidelines for vegetables [35, 36]. Meal costs attribute levels were based on average expenditure from the 2015–16 Household Food Expenditure Survey [37]. To ensure that there was a consistent understanding of each attribute and attribute level, participants were asked to read a short piece of text describing the attributes before completing the DCE (Additional File 3).

### Survey

The internet-based survey collected information on DCE perceptions, dietary intakes, sociodemographic characteristics and health and health behaviours. Adaptive questions were used where appropriate, with conditional display of questions based on responses to other items (e.g. if participants did not report doing any vigorous physical activity then they were not asked how much time was spent doing vigorous activity).

#### DCE perceptions

Following the DCE, participants were asked about their typical weekday meals at home, in relation to preparation time (mins), cost per person (Australian dollar), taste (sufficient; good; very good), nutrition content (low; adequate, optimal) and familiarity (not very; somewhat; very) of the meal. These questions were used to identify what are known as “revealed” preferences, to allow comparison of revealed preferences with stated preferences (from the DCE). Given that some questions were ordinal and some were continuous, these were re-coded into binary variables to ensure consistency of interpreting interaction analyses. Cut points were selected based on sample sizes of binary levels and comparability with DCE attribute levels: nutrition content (low and adequate vs. optimal); cost (≤$10 vs. >$10); taste (sufficient and good vs. very good); familiarity (not very and somewhat vs. very); time (≤20mins vs. > 20 mins). Open-ended questionnaire items were included to collect information on how easy participants found the DCE questions to answer. These included “Were there any characteristics of a meal that are important to you that were not included in these meal options? If yes, please explain what these characteristics were and why they are important to you”. Based on the small amount of resulting qualitative data, these are presented as descriptive information and were not analysed.

#### Dietary intakes

Information on intake of fruit (serves/day), vegetables (serves/day) [38], and discretionary foods and drinks (frequency consumed per month; confectionary, cake, pies, fast food, pizza, hot chips, meat products, milk, flavoured milk, soft drinks, fruit juice, beer, cider, red wine, white wine, port and spirits) were collected using three previously tested questionnaire items [39, 40]. Responses recorded as frequencies per month were converted to daily serve equivalents [41]. Serves per day of fruits and vegetables and total discretionary foods and drinks were reported. Participants were grouped according to whether they met Australian recommendations for fruit and vegetables (≥7 serves/day) and discretionary foods and beverages (≤2.5 serves/day for females and 3 serves/day for males) [36, 42].

#### Sociodemographic characteristics

Age, sex, place of birth (Australian, other), highest educational attainment, individual weekly income, current occupation type, relationship status and living situation were collected in the online survey using previously tested questionnaire items [43]. Education and income were recoded into binary variables for the interaction analyses. Education was recoded into low (no formal qualifications, year 10 or equivalent, year 12 or equivalent, trade/apprenticeship, certificate/diploma) or high (University degree, higher University degree). Income was recoded as low (no income, $1–$119 per week, $120–$299 per week, $300–$499 per week) or high ($500–$699 per week, $700–$999 per week, $1000–$1499 per week, $1500 or more per week). Occupation was categorised as manager/professional, trade/service, clerical/sales, machinery/labourer, student, no paid job. Information on relationship status (in a relationship or not) and living situation (living by myself, with parents/family, with partner/spouse, with flatmates/friends) were collected.

#### Health and health behaviours

Self-reported health, weight status, smoking habits, physical activity and sleep duration were collected using previously tested questionnaire items [43, 44]. Self-reported health was reported as excellent or very good, good, fair or poor. Body mass index (BMI) was estimated from self-reported weight (kg) and height (m). Participants were categorised into non-overweight/obese (BMI < 25 kg/m^2^) and overweight or obese (BMI ≥ 25 kg/m^2^) for the interaction analyses based on WHO classifications [45]. Participants reported their smoking habits (never smoked, ex-smoker and current smoker). Physical activity was assessed using the validated 4-item short International Physical Activity Questionnaire [44]. Minutes of activity per week were calculated by summing the minutes of moderate intensity physical activity and twice the number of minutes spent participating in vigorous intensity physical activity [44]. For the purpose of the descriptive analyses, participants were categorised according to whether they met the physical activity guidelines of at least 150 min of activity per week [46]. Participants self-reported the numbers of hours of sleep on a weekday, and were categorised into whether they met the sleep duration guidelines of between 7 and 9 h per night (No/Yes) for descriptive purposes [47].

### Statistical analyses

Analyses were conducted in R and Stata (Version SE 15.0). Descriptive analyses included mean (SD) for continuous variables and number (%) for categorical variables. Conditional logit models were used for the analysis of the DCE [48]. Conditional logit models are suitable for modelling choice situations in which there are multiple options but only one can be selected. This method models the choice among alternative possible choices as a function of the characteristics of the alternatives, which makes this approach ideally suited to the DCE setting presented [49]. Given that each analysed participant completed 12 choice sets, robust standard errors were specified to account for clustering within individuals. The following utility function was estimated:

$$ \mathsf{V}=\beta \mathsf{1}\times \mathsf{TASTE}\_\mathsf{Good}+\beta \mathsf{2}\times \mathsf{TASTE}\_\mathsf{Very}\_\mathsf{Good}+\beta \mathsf{3}\times \mathsf{TIME}\_\mathsf{15}+\beta \mathsf{4}\times \mathsf{TIME}\_\mathsf{30}+\beta \mathsf{5}\times \mathsf{NUTRITION}\_\mathsf{CONTENT}\_\mathsf{Adequate}+\beta \mathsf{6}\times \mathsf{NUTRITION}\_\mathsf{CONTENT}\_\mathsf{Optimal}+\beta \mathsf{7}\times \mathsf{COST}\_\mathsf{10}+\beta \mathsf{8}\times \mathsf{COST}\_\mathsf{15}+\beta \mathsf{9}\times \mathsf{FAMILIARITY}\_\mathsf{Somewhat}+\beta \mathsf{1}\mathsf{0}\times \mathsf{FAMILIARITY}\_\mathsf{Very} $$

where V represents the observable utility that respondents have for a meal alternative and β1–10 are variable weights (coefficients) linearly associated with each attribute of the DCE. The reference levels for the attributes were as follows: taste (sufficient), time (5 min), nutrition content (low), cost ($5 per person), familiarity (not very). The sign of the coefficient indicates whether the attribute level had a positive or negative effect on the meal choice, relative to the reference category. Preference coefficients were translated to relative importance percentage by dividing the (absolute) difference in utility between the highest and lowest level for a single attribute by the sum of the (absolute) differences of all attributes [50]. A Relative Importance Score between 1 and 5 was assigned to each attribute based on the relative importance percentage. Interaction effects for revealed preferences, sex, education, income, weight status and meeting fruit and vegetable recommendations were added into separate models to investigate their impact on each meal attribute level.

### Sensitivity analyses

Sensitivity analyses were conducted to assess the impact of participants opting out by choosing the “No” option, i.e. that they would not choose to eat that meal. Preference weights were examined with the inclusion of “none” as a meal option. Binary logistic regression models were used to estimate odds ratios (95% CI) of opting out (0 = completed all choice sets, 1 = opted-out at least once) in at least one of the choice sets according to socio-demographic characteristics, health-related behaviours and BMI.

## Results

As shown in Additional File 2, of the 3843 individuals who clicked on the CHOICE survey link, 149 proceeded to the survey after reading the plain language statement and 92 eligible individuals (mean age: 23.9 (SD 3.4) years) completed the survey. The completion rate was 62%. Of the participants who completed the survey, the average time spent completing the survey was 37.6 (SD 141.1) min. On average, 6.04 (SD 12.2) min was spent completing the DCE and 12.09 (SD 9.87) min completing the questionnaire. Most participants were female (61%) and were born in Australia (71%). As shown in Table 1, participants were predominantly in the high income category (64%), were in a managerial/professional position (29%) or were a student (29%). Participants reported mean daily serves of fruit and vegetables of 2.4 (SD 1.5) and 4.8 (SD 1.6), respectively, with 42% of participants meeting recommendations for fruit and vegetables. Mean daily serves of discretionary foods was 13.4 (SD 7.0), with no participants meeting recommendations for discretionary foods. Fifteen percent of participants were current smokers and the majority were not overweight/obese (75%), met physical activity guidelines (71%), sleep guidelines (69%) and had ‘excellent or very good’ health (60%).

**Table 1:** Demographic characteristics and dietary and health-related behaviours of young Australian adults included in the CHOICE study (*n* = 92)

Characteristic	N (%)
Age (years), Mean ± SD	23.9 ± 3.4
Female	56 (60.9)
Born in Australia	65 (70.7)
Education^a^	
Low or middle	46 (50.0)
High	46 (50.0)
Income^b^	
Low	33 (35.9)
High	59 (64.1)
Occupation	
Manager/Professional	27 (29.4)
Clerical/Sales	20 (2.7)
Machinery/Labourer/Trade/Service	13 (14.1)
Student	27 (29.4)
No paid job	5 (5.4)
In a relationship	33 (35.9)
Living situation	
Living with parents/family	32 (35.9)
Living by myself	12 (13.0)
Living with partner/spouse	19 (21.7)
Living with flatmates/friends	27 (29.4)
Meeting dietary guideline recommendations	
Fruit and vegetables (≥7 serves/day)	39 (42.4)
Discretionary foods and beverages (≤2.5–3 serves/day)	0 (0.0)
Smoking status	
Never smoked	63 (68.5)
Ex-smoker	15 (16.3)
Current smoker	14 (15.2)
Overweight (BMI ≥ 25 kg/m^2^)	23 (25.0)
Meet physical activity guidelines (≥150 min of activity/week)	65 (70.7)
Meet sleep duration guidelines (7–9 h/night)	63 (68.5)
Self-reported health	
Excellent or very good	55 (59.8)
Good	31 (33.7)
Fair or poor	6 (6.52)

*BMI* Body Mass Index

^a^ Education: low or medium (no formal qualifications, year 10 or equivalent, year 12 or equivalent, trade/apprenticeship, certificate/diploma) and high (University degree, higher University degree)

^b^ Income: low (no income, $1–$119 per week, $120–$299 per week, $300–$499 per week) or high ($500–$699 per week, $700–$999 per week, $1000–$1499 per week, $1500 or more per week)

### Stated preferences

As shown in Table 2, nutrition content, cost, taste, familiarity and preparation time significantly influenced young adults’ meal choices. The perceived value of a meal was higher when it was higher in nutrition content, tasted better and was more familiar. In contrast, the perceived value of a meal was lower when it took longer to prepare and cost more. The relative importance, based on ranking of attribute coefficients for highest versus lowest levels, indicated that nutrition content was the most important consideration for meal choices, followed by cost, taste, familiarity and time. This ranking was consistent with or without the inclusion of the opt-out option (Table 2).

**Table 2:** Stated preference weights for attributes of a typical weekday meal in young Australian adults (18–30 years) in the CHOICE Study (*n* = 92)

Attribute	Attribute level	Including the opt-out option				Excluding the opt-out option			
		Coefficient	(95% CI)	*P* value	RIS	Coefficient	(95% CI)	*P* value	RIS
Nutrition content	Low (reference)								
	Adequate	0.77	(0.50, 1.04)	< 0.001	1	0.63	(0.37, 0.88)	< 0.001	1
	Optimal	1.26	(0.94, 1.59)	< 0.001		1.11	(0.81, 1.41)	< 0.001	
Cost	$5 per person (reference)								
	$10 per person	− 0.17	(− 0.36, 0.02)	0.083	2	− 0.16	(− 0.35, 0.03)	0.09	2
	$15 per person	− 0.57	(−0.85, − 0.30)	< 0.001		− 0.50	(− 0.75, − 0.24)	< 0.001	
Taste	Sufficient (reference)								
	Good	0.26	(0.05, 0.47)	0.017	3	0.20	(− 0.01, 0.42)	0.06	3
	Very good	0.45	(0.18, 0.72)	0.001		0.38	(0.12, 0.63)	0.004	
Familiarity	Not very (reference)								
	Somewhat	0.18	(0.02, 0.35)	0.026	4	0.13	(− 0.02, 0.28)	0.09	4
	Very	0.39	(0.21, 0.57)	< 0.001		0.37	(0.21, 0.54)	< 0.001	
Time	5 min (reference)								
	15 min	− 0.04	(− 0.20, 0.13)	0.64	5	− 0.06	(− 0.22, 0.10)	0.45	5
	30 min	− 0.37	(− 0.60, − 0.14)	0.002		− 0.33	(− 0.53, − 0.12)	0.002	

Data are dummy coded conditional logit model coefficients and 95% CI. Preference weights indicates utilities for a given attribute level. The RIS is based on the ranking of coefficient absolute values. RIS, Relative Importance Score

### Subgroup interactions

Interactions between meal attributes and demographics (sex, education level and income) and health characteristics (weight status and meeting fruit and vegetable recommendations) are shown in Additional Files 4 and 5, respectively. Female participants and those with higher education had higher preferences for better nutrition content, taste and familiarity compared to males and those with lower education. Females also had a lower preference for higher cost meals than males. Participants who met fruit and vegetable recommendations had a higher preference for better nutrition content than those who didn’t meet recommendations. There was little evidence of interactions of meal attributes with income or weight status.

### Revealed preferences

Following completion of the DCE, young adults provided revealed preference data on attributes of their typical weekday meal at home. The average time preparing a meal was 25.1 (SD 19.3) mins and the average expenditure on a meal was $14.7 (SD 17.6) per person. The majority of young adults reported that their typical weekday meal at home would have a ‘very good’ taste (58%), an ‘adequate’ nutrition content (45%), and would be ‘very familiar’ (51%). Interactions between revealed preferences and attribute levels for stated preferences are shown in Table 3. Participants with a revealed preference for optimal nutrition content, higher cost and very good taste showed higher stated preferences for these attribute levels. In contrast, revealed preferences for familiarity and time were not significantly consistent with the stated preferences.

**Table 3:** Interactions between revealed preference and attribute levels in young Australian adults (18–30 years) in the CHOICE Study excluding the opt-out option (*n* = 92)

Attribute	Attribute level	Coefficient	(95% CI)	*P* value
Nutrition content	Low (reference)			
	Adequate	1.59	(0.92, 2.25)	< 0.001
	Optimal	2.31	(1.58, 3.04)	< 0.001
Cost	$5 per person (reference)			
	$10 per person	0.45	(0.06, 0.83)	0.023
	$15 per person	0.75	(0.26, 1.24)	0.003
Taste	Sufficient (reference)			
	Good	0.63	(0.22, 1.05)	0.003
	Very good	0.69	(0.20, 1.18)	0.006
Familiarity	Not very (reference)			
	Somewhat	0.20	(−0.10, 0.50)	0.20
	Very	0.19	(−0.13, 0.52)	0.24
Time	5 min (reference)			
	15 min	−0.11	(− 0.44, 0.22)	0.51
	30 min	− 0.16	(−0.58, 0.26)	0.46

Data are dummy coded conditional logit model coefficients and 95% CI for the interaction terms from models containing main effects of attribute levels and revealed preferences, and their interactions. Coefficients represent the estimated difference in attribute level coefficients from the DCE between levels of the binary revealed preference moderator. Revealed preferences were as follows, with reference categories listed first: nutrition content (low and adequate vs. optimal); cost (≤$10 vs. >$10); taste (sufficient and good vs. very good); familiarity (not very and somewhat vs. very); time (≤20mins vs. > 20 mins)

### Self-reported influences and sensitivity analyses

**Fig. 2:**
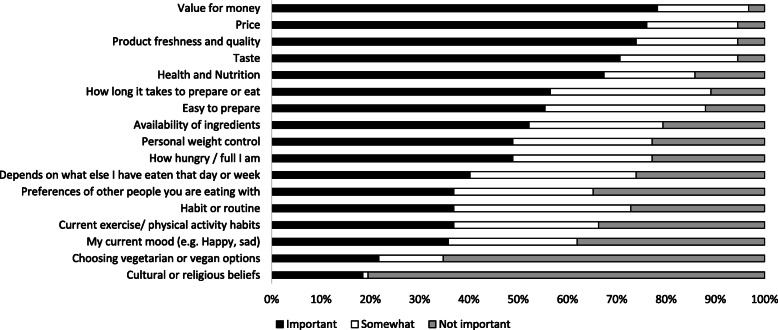
Self-reported barriers to eating a healthy diet in young adults from the CHOICE Study presented in order of importance (*n* = 92)

Self-reported information on the importance of influences on healthy eating were collected as part of the online survey. The most important influences on preparing a healthy meal were value for money (rated as ‘Important’ by 72% of participants), price (70%), product freshness and quality (68%), taste (65%) and health and nutrition (62%; Fig. 2). A total of 45 participants (49%) did not opt out of any choice sets. Of those who opted out of one or more choice sets, about half opted out of 4 sets or less (47%). Odds of opting out were lower in older individuals, females, individuals in relationships and those who met sleep duration recommendations. Odds of opting out were higher in ex-smokers than non-smokers (Additional File 6).

### Qualitative information

Qualitative information on the DCE was collected (Additional File 7). Sixteen participants provided responses on additional attributes of a meal that were important to them that were not included in the meal options. Responses were grouped according to common themes: preferences of others (“Partner preferences”), food preparation factors (e.g. “Cooking method - baking, stove, or raw”), specific health and nutrition related factors (“How filling it is - It’s important that a meal fill you up so you aren’t left unsatisfied and hungry”).

## Discussion

This study aimed to rank influences on young adults’ meal preferences using a DCE. The main findings were that the nutrition content of a meal was ranked as the most important influence on young adults’ meal choice when considered alongside cost, taste, preparation time and familiarity. Cost was the second most important, followed by taste, familiarity and preparation time. Moreover, meal preferences varied according to sex, education and fruit and vegetable intake, where female participants and those with higher education had higher preferences for better nutrition content, taste and familiarity, females had a higher preference for lower cost meals and participants who met fruit and vegetable recommendations had a higher preference for better nutrition content. Confirming previous research in young adults [51], these findings suggest that the focus of dietary interventions in this age group may benefit from being tailored to specific population groups.

Previous observational research suggests that the high palatability, low cost and low preparation time of unhealthy foods are key influences on healthy eating in young adults [2, 6, 33]. Nonetheless, the finding that nutrition content is the most important influence on meal preferences is consistent with other DCEs. The only other DCE on this topic in young adults, a study in 130 undergraduate college students, showed that snacks were chosen primarily based on healthfulness, followed by price, taste, and convenience [18]. Moreover, recent DCEs on meal choices in middle-aged and older adults showed ‘healthiness’ was ranked the most important attribute of a meal [9, 23, 52]. Kershaw et al. [23] showed ‘healthfulness’ was the most important influence on meal choices in a sample of 228 middle-aged adults (mean age 34 years), while Kamphuis et al. [9] showed the same result in a sample of 399 older adults (mean age 63 years). Notably, the definition of nutrition content or healthiness has varied across the literature, with most studies not providing a quantitative definition based on dietary guidelines [9, 21]. In the present study, nutrition content was designed to reflect both the quality and quantity of vegetable serves [35, 36]. Thus, future research should extend this research to investigate a nutrition content definition based on other components of a meal, such as serves of lean meats or alternatives and grain foods and the level of processing.

It is possible that a stated preference for nutrition content was a proxy for a desire to improve health and wellbeing. This is consistent with the existing nutrition literature and is likely to be due to the highly educated and female-skewed sample and the potential for social desirability bias [53]. In a recent systematic review, key enablers of healthy eating included gender-based (female) interest in and implementation of a healthy diet, desire for improved health and for weight management [6]. The potential influence of social desirability in the present study is supported by differences between the stated and revealed preferences of participants. While stated preferences suggested that nutrition content was the most important influence on meal choice, revealed preferences showed that participant’s typical meal would have the highest level of taste (‘very good’) and a lower level of nutrition content (‘adequate’). This is consistent with research showing that healthy eating intentions do not always translate into healthy eating behaviours [54]. Nonetheless, participants who did meet recommendations prioritised nutrition content more, suggesting that stated preferences were comparable with reported dietary behaviours.

Consistent with previous DCEs [9, 23], moderation analyses provided evidence of differences in preferences by participant characteristics. This DCE was the first to investigate differences in stated preferences by income, weight status and dietary intake. Given the observed differences in preferences for nutrition content, cost, taste and familiarly by sex, education level and fruit and vegetables intake, there is potential for tailoring strategies to better address the barriers and enablers of behaviour change in young adults [55]. Notably, developing messages focused on nutrition content for use in young adults who don’t prioritise nutrition content when making eating decisions may not be the most effective strategy for achieving behaviour change. The present findings provide quantitative information on ranking of influences that suggests that while focusing on nutrition content may be helpful for females and higher educated young adults, tailoring dietary interventions and polices to other motivators may be more effective in males and adults with lower levels of education. This is consistent with previous research, where males are more likely to experience greater ambivalence towards nutrition [8]. Moreover, there may be other motivators for healthy eating in young males, such as sports and performance goals, which may require further examination [56]. Alternatively, research suggests that effective motivators could also involve exposing manipulation strategies employed by junk food marketing [57] and targeting ethical and environmental motivators to eat fresh and minimally processed local produce [58].

The present study provides information on the feasibility of using a DCE methodology for understanding food choice behaviours. Participants were less likely to opt out of a DCE meal choice if they were older and female, which is potentially due to higher social desirability in these groups [59, 60]. Consistent with previous research, the present study shows that there are minimal differences in preference coefficients between a forced and a non-forced DCE, showing the feasibility of using both designs [61]. We observed that participants who were more likely to opt out were comparable to those who are more likely to drop out of RCTs, i.e. younger and male [62], although there were no differences by weight status or education level. Moreover, insights from the qualitative data suggested that future DCEs in young adults should consider the role of social influences and cooking skills, which are known determinants of food choice [63, 64].

The present study had a number of limitations that should be acknowledged. This exploratory study aimed to investigate eating behaviours in a self-selected sample of young adults, which resulted in a sample skewed towards more females, highly educated individuals, and those with good overall health. As participants knew the overall goal of the study and that females have a greater desire for improved health and for weight management, the potential influence of social desirability on our findings cannot be discounted. Nonetheless, the present study informs future DCEs in young adults by showing the feasibility of the online design. In addition, it was not possible to include more than five meal characteristics and difference ranges of attribute levels. Thus, there may be other influences on healthy eating not investigated in this study that are also important, and the choice of attribute levels may have impacted the ranking of these influences. However, the present attributes and levels were based on a review of the literature and piloting and the online survey confirmed that these five characteristics were rated the most important among 17 potential influences examined. Future research should consider piloting the use of the attributes and levels in the target group prior to designing the DCE. Furthermore, a more comprehensive definition of nutrition content, based on more than just vegetable intake, would provide further insights into nutrition preferences in young adults. Given the small sample size of this exploratory study, the interactions may be underpowered and thus interpretation should be a balance of effect estimates, CIs and *p*-values. No adjustment for multiple comparisons was included as although multiple tests for interactions were included, this may increase risk of type 2 error [65].

The present study had a number of strengths. A key strength of this study is its novelty, as this is the first study to examine meal preferences in young adults using a quantitative DCE. Definitions of meal attributes and levels were provided to help minimise subjectivity of how meal attributes were understood by participants. In addition, the dual none response option used in the present study enabled a comparison of participant characteristics between those who opted out of DCE meal choices and those who did not. The exploratory examination of interactions between stated and revealed preferences as well as with demographic and health characteristics provides further information to inform effective tailoring of dietary behaviour change strategies.

In conclusion, nutrition content was the most important influence on meal choice in this sample of young adults. Cost, taste, familiarity and preparation time also influenced meal choices. The impact of nutrition content, cost, taste and familiarity on meal preferences varied according to sex, education level and fruit and vegetable intake, suggesting that the focus of dietary interventions in young adults may benefit from being tailored to specific population groups. Future DCEs should include more male participants and a greater diversity in socio-economic position, as well as considering the impact of social desirability bias on stated preferences.

## Supplementary Information

**Additional file 1.** Checklist for Reporting Results of nutritional epidemiology - STROBE-nut guidelines.

**Additional file 2.** Flow diagram of participants included in the CHOICE study.

**Additional file 3.** Meal attribute definitions provided to young adult participants in the online survey.

**Additional file 4. **Interactions between stated preferences for attributes and sex, education and income in the CHOICE Study excluding the opt-out option (*n* = 92).

**Additional file 5. **Interactions between stated preferences for attributes and overweight status and meeting fruit and vegetable recommendations in the CHOICE Study excluding the opt-out option (*n* = 92).

**Additional file 6. **Odds ratio (95% CI) of opting out (0 = completed all, 1 = opted-out) in at least one of the choice sets according to socio-demographic characteristics, health-related behaviours and BMI of young adults from the CHOICE Study (*n* = 92).

**Additional file 7. **Verbatim responses when asked which additional meal attributes should have been included in the discrete choice experiment (*n* = 16).

## Data Availability

The datasets generated and/or analysed during the current study are not publicly available due ethical restrictions but are available from the corresponding author on reasonable request.

## Abbreviations

BMI: Body mass index
DCE: Discrete choice experiment
