# Supplementary material for: Ranking of meal preferences and interactions with demographic characteristics: a discrete choice experiment in young adults
Source: Int J Behav Nutr Phys Act. 2020 Dec 1;17:157. doi: 10.1186/s12966-020-01059-7 (PMC7708905; doi:10.1186/s12966-020-01059-7)
Supplement: Supplementary file 2 — Additional file 2. Flow diagram of participants included in the CHOICE study. [file 12966_2020_1059_MOESM2_ESM.docx]

Individuals who clicked on the CHOICE survey link

*n* = 3,843

Entered the survey

*n* = 149

EXCLUDED, *n* = 3,694

- Did not enter the survey after reading the PLS

EXCLUDED, *n* = 57

- Ineligible, *n* = 23
- No consent, *n* = 5
- Dropped out, *n* = 29

Data available for analysis

*n* = 92

**Supplementary Figure 1.** Flow diagram of participants included in the CHOICE study
