# Supplementary material for: Ranking of meal preferences and interactions with demographic characteristics: a discrete choice experiment in young adults
Source: Int J Behav Nutr Phys Act. 2020 Dec 1;17:157. doi: 10.1186/s12966-020-01059-7 (PMC7708905; doi:10.1186/s12966-020-01059-7)
Supplement: Supplementary file 3 — Additional file 3. Meal attribute definitions provided to young adult participants in the online survey. [file 12966_2020_1059_MOESM3_ESM.docx]

**Supplementary Table 2.** Meal attribute definitions provided to young adult participants in the online survey

| Attribute | Level | Definition |
| --- | --- | --- |
| Nutrition content^1^ | Low | Does not contain any vegetables |
|  | Adequate | Contains a serve of canned vegetables (with added sugar or salt), pickled vegetables or vegetables roasted in oil or marinated.  A serve is about 75g (e.g. half a cup of cooked green or orange vegetables) |
|  | Optimal | Contains one or more serves of fresh, frozen or canned vegetables (without any added salt or sugar). |
| Taste | Sufficient | The meal is not very enjoyable to eat but you may tolerate it to get another benefit. |
|  | Good | The meal is somewhat enjoyable to eat |
|  | Very good | The meal is very enjoyable to eat and gives you a lot of satisfaction. |
| Familiarity | Not very | You never or rarely eat the meal |
|  | Somewhat | You eat this meal at least once a month but less than once per week |
|  | Very | You eat this meal at least once a week |

1, Attribute levels for nutrition content are based on the quality and quantity of vegetables in a meal in accordance with the Australian Dietary Guidelines (1) and the Meat and Livestock Healthy Meal Guide. (2)

**References**

1. Australian Government National Health and Medical Research Council Department of Health and Ageing. Eat for Health. Australian Dietary Guidelines 2013 Accessed 21 October 2016. Available from: <https://www.eatforhealth.gov.au/sites/default/files/files/the_guidelines/n55_australian_dietary_guidelines.pdf>.

2. Meat & Livestock Australia. Healthy Meal Guide 2015 Accessed 15 May 2018. Available from: [https://www.mlahealthymeals.com.au/#](https://www.mlahealthymeals.com.au/).
