# Supplementary material for: Ranking of meal preferences and interactions with demographic characteristics: a discrete choice experiment in young adults
Source: Int J Behav Nutr Phys Act. 2020 Dec 1;17:157. doi: 10.1186/s12966-020-01059-7 (PMC7708905; doi:10.1186/s12966-020-01059-7)
Supplement: Supplementary file 4 — Additional file 4. Interactions between stated preferences for attributes and sex, education and income in the CHOICE Study excluding the opt-out option (n = 92). [file 12966_2020_1059_MOESM4_ESM.docx]

**Supplementary Table 3**. Interactions between stated preferences for attributes and sex, education and income in the CHOICE Study excluding the opt-out option (n=92).

| Attribute | Attribute level | Sex | | | Education level | | | Income | | |
| --- | --- | --- | --- | --- | --- | --- | --- | --- | --- | --- |
|  |  | Coefficient | (95% CI) | P value | Coefficient | (95% CI) | P value | Coefficient | (95% CI) | P value |
| Nutrition content | Low (ref) |  |  |  |  |  |  |  |  |  |
|  | Adequate | 0.43 | (-0.09, 0.95) | 0.11 | 0.44 | (-0.11, 0.99) | 0.12 | -0.16 | (-0.77, 0.45) | 0.62 |
|  | Optimal | 0.96 | (0.39, 1.54) | 0.001 | 0.82 | (0.18, 1.46) | 0.012 | -0.28 | (-0.95, 0.40) | 0.43 |
| Cost | $5 per person (ref) |  |  |  |  |  |  |  |  |  |
|  | $10 per person | -0.21 | (-0.61, 0.18) | 0.29 | 0.13 | (-0.25, 0.52) | 0.50 | 0.15 | (-0.26, 0.56) | 0.47 |
|  | $15 per person | -0.55 | (-1.05, -0.04) | 0.033 | -0.06 | (-0.57, 0.45) | 0.82 | 0.46 | (-0.09, 1.01) | 0.10 |
| Taste | Sufficient (ref) |  |  |  |  |  |  |  |  |  |
|  | Good | 0.32 | (-0.11, 0.76) | 0.15 | 0.25 | (-0.16, 0.67) | 0.23 | 0.08 | (-0.37, 0.53) | 0.71 |
|  | Very good | 0.60 | (0.09, 1.11) | 0.021 | 0.70 | (0.19, 1.21) | 0.008 | 0.34 | (-0.18, 0.86) | 0.20 |
| Familiarity | Not very (ref) |  |  |  |  |  |  |  |  |  |
|  | Somewhat | 0.38 | (0.07, 0.68) | 0.016 | 0.26 | (-0.05, 0.56) | 0.10 | -0.04 | (-0.35, 0.26) | 0.80 |
|  | Very | 0.41 | (0.10, 0.73) | 0.010 | 0.34 | (0.00, 0.68) | 0.048 | -0.01 | (-0.35, 0.33) | 0.95 |
| Time | 5 minutes (ref) |  |  |  |  |  |  |  |  |  |
|  | 15 minutes | -0.18 | (-0.51, 0.15) | 0.29 | 0.07 | (-0.26, 0.40) | 0.69 | -0.25 | (-0.55, 0.06) | 0.11 |
|  | 30 minutes | -0.30 | (-0.72, 0.12) | 0.16 | -0.05 | (-0.47, 0.37) | 0.82 | -0.17 | (-0.60, 0.27) | 0.45 |

Data are dummy coded conditional logit model coefficients and 95% CI for the interaction terms from models containing main effects of attribute levels and moderators, and their interactions. Coefficients represent the estimated difference in attribute level coefficients from the DCE between levels of the binary moderator. Males, low education and low income were used as the reference categories. Education: low (no formal qualifications, year 10 or equivalent, year 12 or equivalent, trade/apprenticeship, certificate/diploma) and high (University degree, higher University degree). Income: low (no income, $1-$119 per week, $120-$299 per week, $300-$499 per week) and high ($500-$699 per week, $700-$999 per week, $1,000-$1,499 per week, $1,500 or more per week)
