# Supplementary material for: Ranking of meal preferences and interactions with demographic characteristics: a discrete choice experiment in young adults
Source: Int J Behav Nutr Phys Act. 2020 Dec 1;17:157. doi: 10.1186/s12966-020-01059-7 (PMC7708905; doi:10.1186/s12966-020-01059-7)
Supplement: Supplementary file 5 — Additional file 5. Interactions between stated preferences for attributes and overweight status and meeting fruit and vegetable recommendations in the CHOICE Study excluding the opt-out option (n = 92). [file 12966_2020_1059_MOESM5_ESM.docx]

**Supplementary Table 4**. Interactions between stated preferences for attributes and overweight status and meeting fruit and vegetable recommendations in the CHOICE Study excluding the opt-out option (n=92).

| Attribute | Attribute level | Overweight status | | | | Meet fruit and vegetable recommendations | | |
| --- | --- | --- | --- | --- | --- | --- | --- | --- |
|  |  | Coefficient | (95% CI) | P value | Coefficient | | (95% CI) | P value |
| Nutrition content | Low (ref level) |  |  |  |  | |  |  |
|  | Adequate | -0.12 | (-0.64, 0.40) | 0.65 | 0.68 | | (0.04, 1.32) | 0.036 |
|  | Optimal | -0.40 | (-1.04, 0.25) | 0.23 | 1.40 | | (0.67, 2.13) | <0.001 |
| Cost | $5 per person (ref level) |  |  |  |  | |  |  |
|  | $10 per person | 0.27 | (-0.20, 0.74) | 0.25 | 0.24 | | (-0.15, 0.62) | 0.23 |
|  | $15 per person | 0.58 | (-0.01, 1.17) | 0.06 | -0.14 | | (-0.67, 0.39) | 0.61 |
| Taste | Sufficient (ref level) |  |  |  |  | |  |  |
|  | Good | 0.31 | (-0.16, 0.79) | 0.20 | 0.08 | | (-0.36, 0.52) | 0.73 |
|  | Very good | 0.20 | (-0.39, 0.79) | 0.51 | 0.20 | | (-0.37, 0.77) | 0.49 |
| Familiarity | Not very (ref level) |  |  |  |  | |  |  |
|  | Somewhat | 0.04 | (-0.34, 0.42) | 0.83 | 0.25 | | (-0.04, 0.55) | 0.09 |
|  | Very | 0.13 | (-0.25, 0.51) | 0.51 | 0.33 | | (0.00, 0.66) | 0.05 |
| Time | 5 minutes (ref level) |  |  |  |  | |  |  |
|  | 15 minutes | 0.04 | (-0.36, 0.43) | 0.86 | 0.01 | | (-0.33, 0.35) | 0.95 |
|  | 30 minutes | 0.27 | (-0.22, 0.76) | 0.28 | 0.08 | | (-0.35, 0.51) | 0.71 |

Data are dummy coded conditional logit model coefficients and 95% CI for the interaction terms from models containing main effects of attribute levels and revealed preferences, and their interactions. Coefficients represent the estimated difference in stated preference coefficients from the DCE between levels of the binary revealed preference moderator, where a larger coefficient indicates a larger agreement between revealed and stated preferences. Overweight status: normal weight or underweight (BMI <25kg/m^2^; reference category) and overweight or obese (BMI ≥ 25kg/m^2^). Meet fruit and vegetable recommendations: yes (≥2 serves of fruit and ≥5 serves of vegetables) and no (<2 serves of fruit and <5 serves of vegetables; reference category)
