# Supplementary material for: Ranking of meal preferences and interactions with demographic characteristics: a discrete choice experiment in young adults
Source: Int J Behav Nutr Phys Act. 2020 Dec 1;17:157. doi: 10.1186/s12966-020-01059-7 (PMC7708905; doi:10.1186/s12966-020-01059-7)
Supplement: Supplementary file 6 — Additional file 6. Odds ratio (95% CI) of opting out (0 = completed all, 1 = opted-out) in at least one of the choice sets according to socio-demographic characteristics, health-related behaviours and BMI of young adults from the CHOICE Study (n = 92). [file 12966_2020_1059_MOESM6_ESM.docx]

| Characteristic | OR (95% CI) | P value |
| --- | --- | --- |
| Age (years) | 0.81 (0.71, 0.93) | 0.003 |
| Female | 0.29 (0.12, 0.69) | 0.006 |
| Born in Australia | 0.78 (0.32, 1.91) | 0.58 |
| Education |  |  |
| Low (ref) |  |  |
| Middle | 1.80 (0.23, 14.1) | 0.58 |
| High | 0.64 (0.08, 4.98) | 0.67 |
| Income |  |  |
| Low (ref) |  |  |
| Middle | 2.12 (0.83, 5.39) | 0.11 |
| High | 1.09 (0.34, 3.46) | 0.89 |
| Occupation |  |  |
| Manager/Professional (ref) |  |  |
| Trade/Service | 2.15 (0.58, 8.00) | 0.25 |
| Clerical/Sales | 1.26 (0.33, 4.73) | 0.74 |
| Machinery/Labourer | 1.62 (0.23, 11.26) | 0.63 |
| Student | 0.86 (0.30, 2.51) | 0.79 |
| No paid job or Other | 0.72 (0.10, 5.01) | 0.74 |
| In a relationship | 0.21 (0.08, 0.53) | 0.001 |
| Living situation |  |  |
| Living by myself (ref) |  |  |
| Living with parents/family | 2.67 (0.69, 10.4) | 0.16 |
| Living with partner/spouse | 0.37 (0.76, 1.83) | 0.22 |
| Living with flatmates/friends | 2.04 (0.51, 0.81) | 0.31 |
| Other | 1.40 (0.07, 28.1) | 0.83 |
| Smoking status |  |  |
| Never smoked (ref) |  |  |
| Ex-smoker | 21.3 (2.63, 172.2) | 0.004 |
| Current smoker | 2.03 (0.63, 6.55) | 0.24 |
| BMI (kg/m^2^) | 1.01 (0.90, 1.14) | 0.86 |
| Meet physical activity guidelines | 0.50 (0.20, 1.27) | 0.15 |
| Meet sleep duration guidelines | 0.27 (0.10, 0.70) | 0.007 |
| Health |  |  |
| Excellent or very good (ref) |  |  |
| Good | 1.09 (0.45, 2.63) | 0.85 |
| Fair or poor | 0.18 (0.02, 1.64) | 0.13 |

**Supplemental Table 5**. Odds ratio (95% CI) of opting out (0=completed all, 1=opted out) in at least one of the choice tasks according to socio-demographic characteristics, health-related behaviours and BMI of young adults from the CHOICE Study (n=92)

BMI, Body Mass Index; Physical activity guidelines were at least 150 minutes of activity per week and sleep duration guidelines were 7-9 hours per night. Not meeting guidelines were the reference categories.
