# Supplementary material for: Ranking of meal preferences and interactions with demographic characteristics: a discrete choice experiment in young adults
Source: Int J Behav Nutr Phys Act. 2020 Dec 1;17:157. doi: 10.1186/s12966-020-01059-7 (PMC7708905; doi:10.1186/s12966-020-01059-7)
Supplement: Supplementary file 7 — Additional file 7. Verbatim responses when asked which additional meal attributes should have been included in the discrete choice experiment (n = 16). [file 12966_2020_1059_MOESM7_ESM.docx]

**Supplementary Table 6**. Verbatim responses when asked which additional meal attributes should have been included in the discrete choice experiment (n=16)^1^

| Preferences of others | Food preparation factors | Nutrition and health related factors |
| --- | --- | --- |
| Level of compromising meal options & taste preferences with my husband | Involves little clean-up | How filling it is - It's important that a meal fill you up so you aren't left unsatisfied and hungry |
| Child friendly | Whether it will allow for leftovers the next day | No sugar |
| Preference of partner/friends | Ease of preparing / storing ingredients | Balance (of protein, veggies and carbs) |
| Has to take into account dietary requirements (e.g. gluten free for me, dairy free for my partner) | Versatility of ingredients/how long they keep (e.g. potatoes or lentils vs bok choy) | Home town taste |
|  | Less time to prepare, tasty, cheap and easy to cook |  |
|  | Cooking method - baking, stove, or raw |  |
|  | Ability to freeze/serving size |  |
|  | Food volume |  |

1, Question: Were there any characteristics of a meal that are important to you that were not included in these meal options? If yes, please explain what these characteristics were and why they are important to you.
